# Population distribution and causes of mortality of smooth-coated otters, *Lutrogale perspicillata*, in Singapore

**DOI:** 10.1093/jmammal/gyad007

**Published:** 2023-03-01

**Authors:** Anusha Shivram, N Sivasothi, Chia-Da Hsu, Karen E Hodges

**Affiliations:** Department of Biology, University of British Columbia Okanagan, Kelowna, British Columbia V1V 1V7, Canada; Department of Biological Sciences, National University of Singapore, Singapore 117558, Singapore; Veterinary Department, Mandai Wildlife Group, 80 Mandai Lake Road, Singapore 729826, Singapore; Department of Biology, University of British Columbia Okanagan, Kelowna, British Columbia V1V 1V7, Canada

**Keywords:** citizen science, human–wildlife conflict, *Lutrogale perspicillata*, roadkill, territorial conflict, urban ecology

## Abstract

Smooth-coated otters (*Lutrogale perspicillata*) were transient in Singapore before one resident family group was observed in 1998, presumably having recolonized from Peninsular Malaysia. A population survey in 2017 revealed a minimum of 11 groups and 79 individuals. Since then, movements of otter groups within urban areas have led to increasing numbers of human–otter encounters, including conflicts. We determined the current abundance, population structure, and distribution of smooth-coated otters in Singapore. We assessed seven sampling zones nationwide through verified sighting records and social media. Mortality records from 2019 to 2021 were sourced from the Otter Working Group and Wildlife Reserves Singapore. In early 2021, there were a minimum of 17 groups and 170 individuals. Groups ranged from 2 to 24 individuals. Smooth-coated otters occupy coastal areas, waterways, reservoirs, and sites within the city center in urban gardens and ponds. Following territorial conflicts at waterways, smooth-coated otter groups moved into the urban matrix. Vehicle collisions are the main cause of mortality and are frequent at dams separating freshwater and coastal habitats. While there is a clear increase in smooth-coated otter numbers since 2017, there remain multiple natural and human-caused threats to otter persistence.

Smooth-coated otters (*Lutrogale perspicillata*) are semiaquatic mammals distributed across South Asia (Khan et al. 2009; Acharya and Lamsal 2010; Narasimmarajan et al. 2021), and Southeast Asia (Dong et al. 2010; Kamjing et al. 2017; Abdul-Patah et al. 2020) with populations in Iraq (Omer et al. 2012). Smooth-coated otters are listed as ‘Vulnerable’ in the IUCN Red List of Threatened Species (2021) as they are threatened by habitat loss, persecution by humans, and wetland and waterway pollution (Acharya and Lamsal 2010; Dong et al. 2010; Nawab and Hussain 2012). Across Asia, these threats are causing marked smooth-coated otter population declines, prompting calls to protect remaining populations (Suthar et al. 2017; Li and Chan 2018; Zhang et al. 2018).

Natural habitats occupied by smooth-coated otters include wetlands and rivers with riparian vegetation (Anoop and Hussain 2004; Dong et al. 2010; Narasimmarajan et al. 2021). Yet, smooth-coated otters have shown resilience to converted landscapes, occupying paddy fields in Malaysia and aquaculture ponds in Thailand (Foster-Turley 1992; Kamjing et al. 2017). Subsidized food resources in urban ponds and waterways provide feeding opportunities in modified landscapes (Kamjing et al. 2017). However, the use of these habitats brings otters in proximity to humans, leading to human–otter conflicts. For example, raiding of aquaculture fishponds have led to the persecution of different species of otters by humans around the world (Myšiak et al. 2004; Kloskowski 2011; Duplaix and Savage 2018).

The island nation Singapore has faced rapid industrialization and urbanization in the past six decades (Tan et al. 2013). The near extirpation of smooth-coated otters in the 1970s and 1980s was attributed to pollution and large-scale coastal land reclamation during this period (Theng and Sivasothi 2016). Efforts to clean and restore waterways in Singapore, and subsequent increases in the availability of prey and suitable habitats have facilitated the return and increase in smooth-coated otter populations since 1998 (Khoo and Lee 2020). In the same period, growing development in Johor, Malaysia, is believed to have displaced otters and encouraged recolonization of Singapore (Theng and Sivasothi 2016; Khoo and Lee 2020). By 2017, there were at least 79 smooth-coated otters in 11 groups in Singapore (Khoo and Sivasothi 2018b).

Smooth-coated otters initially recolonized protected mangrove and coastal habitats in Sungei Buloh Wetland Reserve in the north region and Pulau Ubin, a northeastern island of Singapore (Baker 2000; Theng and Sivasothi 2016) but subsequently spread to multiple urban waterways including the densely populated city center of Singapore in the south (Theng and Sivasothi 2016; Khoo and Sivasothi 2018b). Smooth-coated otters in Singapore use canals and reservoirs close to humans (Khoo and Sivasothi 2018b) as has also been observed in India (Anoop and Hussain 2004; Khan et al. 2014). In 2019, smooth-coated otters were observed raising pups in artificial lentic waterbodies in Singapore Botanic Gardens located away from main waterways within residential and commercial city areas (Ng 2020; Turrell 2020) highlighting the increasing tolerance of urban smooth-coated otter groups to humans.

Smooth-coated otters have social groups that generally consist of a parental pair accompanied by multiple litters (Hussain 1996). Smooth-coated otter groups maintain core territories with holts (dens), grooming, and foraging sites that they mark and defend against conspecific groups (Hussain 1993). At the Central Watershed of Singapore, located at the city center, territoriality between the ‘Bishan’ and ‘Marina’ groups have been well-documented by members of the public and in previous studies (Fast Snail 2016; Khoo and Sivasothi 2018a; Lay 2020). Agonistic interactions include vocalizations, pursuit, and physical fights that have resulted in pup mortality (Khoo and Sivasothi 2018a, 2018b). As of 2020, other otter groups have been observed in conflict with Bishan and Marina at the Central Watershed, indicating changes to the territorial landscape (Zheng 2020).

Direct and indirect human encounters with smooth-coated otters have prompted debate about the management of otters in Singapore (Lee 2020; Pierson 2020). Smooth-coated otters within the city center have damaged property when foraging in fishponds in residential and commercial areas (Pierson 2020; Tham 2021). The proximity of smooth-coated otters to human-dominated habitats in Singapore can also negatively impact otter survival. For example, smooth-coated otters have died as a result of vehicle collisions and injuries from entanglement in unattended fishing traps (Khoo and Sivasothi 2018a, 2018b; Tan 2019a). Exploring the causes of mortality to smooth-coated otters in Singapore can shed light on the influence of urban environments on their survival.

The Otter Working Group (OWG), formed in 2013, consists of community members and researchers, as well as governmental, corporate, and nongovernmental entities (Khoo and Lee 2020). The OWG works collaboratively to manage human–otter encounters, facilitate public engagement, and conduct otter rescues and release (Khoo et al. 2019). Community members in the working group known as ‘otter-watchers’ monitor smooth-coated otters in Singapore and are akin to ‘serious birders’ as defined by Cole and Scott (1999). Beyond simply observing otters, they contribute as citizen scientists, student mentors, and members of the IUCN/SSC Otter Specialist Group.

Surveying otters can be challenging given their cryptic behaviors, semiaquatic lifestyle, and extensive home ranges (Boitani et al. 2012, Kruuk 2011). As such, smooth-coated otter populations are monitored by indirect survey methods including track and sign surveys, camera traps, and interviews with residents (Khan 2015; Kamjing et al. 2017; Palei et al. 2020). While track and sign surveys provide information on the presence of otters, they are limited in their ability to reveal population structure (Yoxon and Yoxon 2014).

In urban Singapore smooth-coated otters move, create spraint (scent-marking) sites, holts, and resting sites in areas accessible to humans (Khoo and Sivasothi 2018b). Their proximity to humans has facilitated the direct observation and monitoring of smooth-coated otter groups by otter-watchers (Theng and Sivasothi 2016; Khoo and Sivasothi 2018b). Otter-watchers who are part of the OWG manage the Facebook groups OtterWatch and OtterCity that have a local and international following of over 49,000 and 177,000 members, respectively, as of September 2021. These otter-watchers compile sighting records on social media chat groups on ‘WhatsApp’ and support queries about current and potential sources of human–otter conflicts as part of OWG management efforts. Although most smooth-coated otters lack individually distinguishable features, the daily to weekly monitoring of multiple smooth-coated otter groups by otter-watchers provides the temporal resolution necessary to distinguish between groups and monitor their changing population structure and distribution. In Singapore, our understanding of otter populations benefits from the sustained documentation by otter-watchers.

With the increasing presence of smooth-coated otters in intensively developed urban environments of Singapore (Khoo and Sivasothi 2018b), there is a pressing need to understand the changes in smooth-coated otter abundance and distribution. Collaborations with otter-watchers across Singapore can facilitate the collation of such data at the scale required. This information will support conservation efforts for the species, as well as help manage human–wildlife conflict. In this study, we applied citizen science to address three objectives relevant to smooth-coated otters within Singapore to: (i) determine the trends in smooth-coated otter distribution, abundance, and population structure since 2017; (ii) document intraspecific conflict and their outcomes at the Central Watershed; and (iii) determine the causes of smooth-coated otter mortality. Collectively, this information gives a current portrait of the status and conservation concerns of smooth-coated otters in Singapore.

## Materials and Methods

### Study area.—

Singapore (1°22ʹN 103°48ʹE) is separated from the southern tip of Peninsular Malaysia by the Johor Straits. Singapore consists of a main island of 728 km^2^ and 62 smaller islands including Pulau Ubin (10.2 km^2^), Sentosa (4.71 km^2^), and Coney Island (1.33 km^2^; Singapore Land Authority 2021). The average population density is 7,688 km^2^ (Department of Statistics Singapore 2022). We worked on Pulau Ubin and the main island, where the landscape is largely urbanized, with less than 5% of primary natural habitat remaining (Brook et al. 2003), although 56% of land is now managed or unmanaged vegetation cover due to catchment protection and greening efforts (Tan et al. 2013). Within the urbanized area, an extensive network of parks and green corridors termed park connectors have been developed since 1991 (Tan 2006). Most parks and park connectors are adjacent to waterways and waterbodies (Tan 2006). Waterways are canalized and dammed rivers, forming an extensive drainage network across Singapore divided into three water catchments, the Central Watershed, Eastern Watershed, and Western Watershed (Public Utilities Board 2014). The Central Watershed encapsulates the downtown core of Singapore where the highest number of employed residents work (Department of Statistics Singapore 2021).

**Fig. 1.**
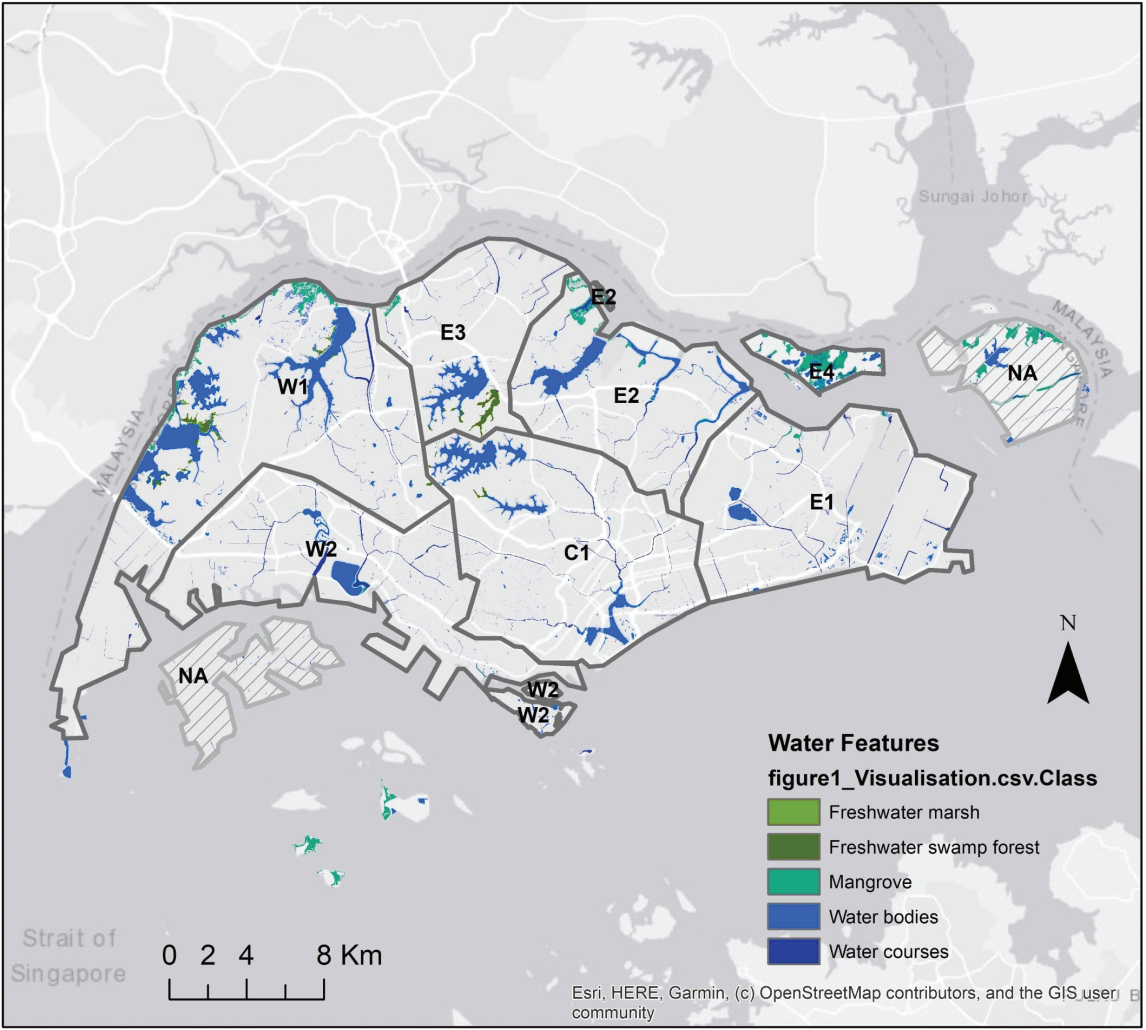
*—*Map of Singapore divided into seven geographic study zones according to the three watersheds; Central Watershed (C1), Eastern Watershed (E1–E4), and the Western Watershed (W1, W2; Public Utilities Board 2014). Prominent water features across Singapore are displayed on the map, sourced from Gaw et al. (2019). Pulau Tekong and Jurong Island (marked NA) were excluded from the study as they are restricted from public access. Number of otter-watchers in each zone are C1 = 4, E1 = 4, E2 = 5, E3 = 0, E4 = 1, W1 = 1, W2 = 2. The World Light Gray Base map was used as a base layer (Esri World Light Gray Base 2011).

We designated seven geographic study zones based on watersheds and main rivers and canals in each watershed (Public Utilities Board 2020; Fig. 1). The urban matrix in this study is defined as the heterogeneous built landscape surrounding reservoirs and waterways that encapsulates ponds in urban gardens, residential, and commercial areas.

### Population abundance and distribution.—

Otter-watchers are community members that monitor smooth-coated otter groups. Four otter-watchers within the OWG monitor otter populations throughout Singapore and communicate with local otter-watchers (e.g., Northeast Otter Network). Otter-watchers use the network of park connectors adjacent to waterways to track otter populations on foot and on bicycle. Otter-watchers use both digital single-lens reflex camera equipment and phone cameras to capture photographs and videos of otters to support otter sighting records shared on WhatsApp messaging chats with other otter-watchers and the OWG.

From 1 September 2020 to 5 March 2021, we consulted 16 otter-watchers to determine information on the populations of smooth-coated otters (Supplementary Data SD1). We obtained the contact information of localized otter-watchers from the OWG and the private WhatsApp group ‘Otterfriends - Singapore.’ WhatsApp was chosen for record submission as it was the preferred communication platform among otter-watchers in Singapore. From each otter-watcher, we requested information on the location of otter group sighted, number of otters, time and date, and photographic or videographic evidence. When there was an observable body size difference between otters in the group, we requested separate counts of pups and adults. Subadults were not distinguished to reduce observer variability. In addition, we recorded comments on intra- and interspecies interactions or unusual smooth-coated otter behavior.

To identify habitat variables at each zone, we conducted field surveys alongside otter-watchers to document latrine sites, holts, and other locations frequented by smooth-coated otters. We categorized smooth-coated otter habitats as coastal, mangrove, reservoir, waterway (canalized or naturalized), and urban ponds. To supplement otter-watcher records and field surveys, we collated sightings posted on public social media groups including Facebook, Instagram, and YouTube, making sure to avoid duplicate photographs or videos across platforms.

### Analysis of sighting records.—

The analysis of smooth-coated otter records to determine the number and distribution of smooth-coated otters was a three-step process to ensure robustness of the data. First, we examined each submission to record the number of otters reported by the observers in each sighting. Additionally, we independently counted the maximum number of otters visible in photographic and video evidence. We recorded the names of groups given by otter-watchers. Lone otters were excluded from the final count due to the inability to distinguish all individual otters (Khoo and Sivasothi 2018b).

**Fig. 2.**
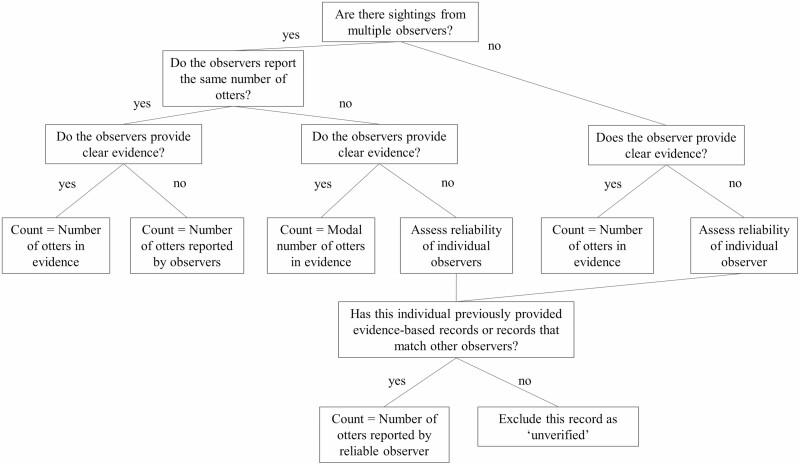
*—*Flowchart used in analysis step 2 to determine the number of smooth-coated otters per group using observer sightings. Observers refer to otter-watchers, independent field surveys, and social media posts. “Clear evidence” refers to unambiguous photographs or video in which otters are clearly distinct and countable.

Second, we determined the reliability of data available for each otter group to find the total count of otters in each group (Fig. 2). Reliability was determined based on the number of observers providing reports and the availability of evidence for each group. For smooth-coated otter groups with only one observer record or multiple reports with differing otter numbers and no clear visual evidence, we determined the count of otters by assessing the reliability of individual observers. To account for variations in observer skills, other projects have weighed volunteer contributions based on their agreement with other observers (Hines et al. 2015) or have obtained ‘vouchers’ (i.e., data that can be validated by experts; Kosmala et al. 2016). In this study, if observers previously provided evidence-based records or their records were confirmed by other observers, we accepted the number of otters reported by the observer. If the observer did not meet either of the two criteria, we excluded that individual record as unverified.

The last step of the analysis of records was to determine if groups were distinct (Khoo and Sivasothi 2018b). We considered two groups as distinct if they were sighted on the same day within one study zone. In addition, if there were differences in the number of pups or if the number of adults differed by more than three, we considered the two groups distinct.

### Territorial conflict and movement of otters.—

To understand intergroup dynamics and movements of smooth-coated otters, we obtained records of four otter groups within the Singapore Central Watershed. We sourced sighting records from January 2020 to August 2020 from three otter-watchers in the OWG, as well as observing otters ourselves and obtaining social media records. We noted the number of behavioral interactions among the otter groups related to territoriality.

To document observable behaviors during territorial conflicts, video-recorded interactions between groups were examined to record instances of vocalization, chase, or physical contact between otter groups. Agonistic interactions were then classified as withdrawal, vocalization, or escalation. Withdrawal occurred when one group approached a holt or waterway occupied by another group, then moved away without further interaction (Erlinge 1968). Vocalization occurred when animals in one or both groups yipped or gave short barks; short chases without physical interaction sometimes also occurred (Leuchtenberger et al. 2015). Escalation included cases where there was physical interaction between animals of the two groups, such as biting, lunges, or wrestling (Khoo and Sivasothi 2018a). We noted if interactions occurred at waterways or within the urban matrix, and recorded locations visited by otters within 3 days of the interactions.

### Causes of mortality for otters.—

Smooth-coated otter mortalities are reported to the OWG through public reports to National Parks Board, Animal Concerns Research and Education Society, OtterWatch, and OtterCity. When carcasses could be retrieved and were not significantly decomposed, the cause of death was verified by necropsy conducted by Mandai Wildlife Group. If necropsies were not conducted, we classified carcasses found on roads as roadkill; the cause of death for carcasses found elsewhere was classified as unknown.

We compiled the causes of smooth-coated otter mortality from 1 January 2019 to 16 August 2021 using information from the OWG and necropsy data made available by Mandai Wildlife Group and National Parks Board. Causes verified by necropsy were distinguished from those that were not. If smooth-coated otters died due to vehicle collision, we analyzed the distance of the collision from a waterway. Roadkill sites were classified into three categories of road—a road that crosses a waterway, a road adjacent to a waterway, or an inland road.

## Results

### Population abundance and structure.—

**Fig. 3.**
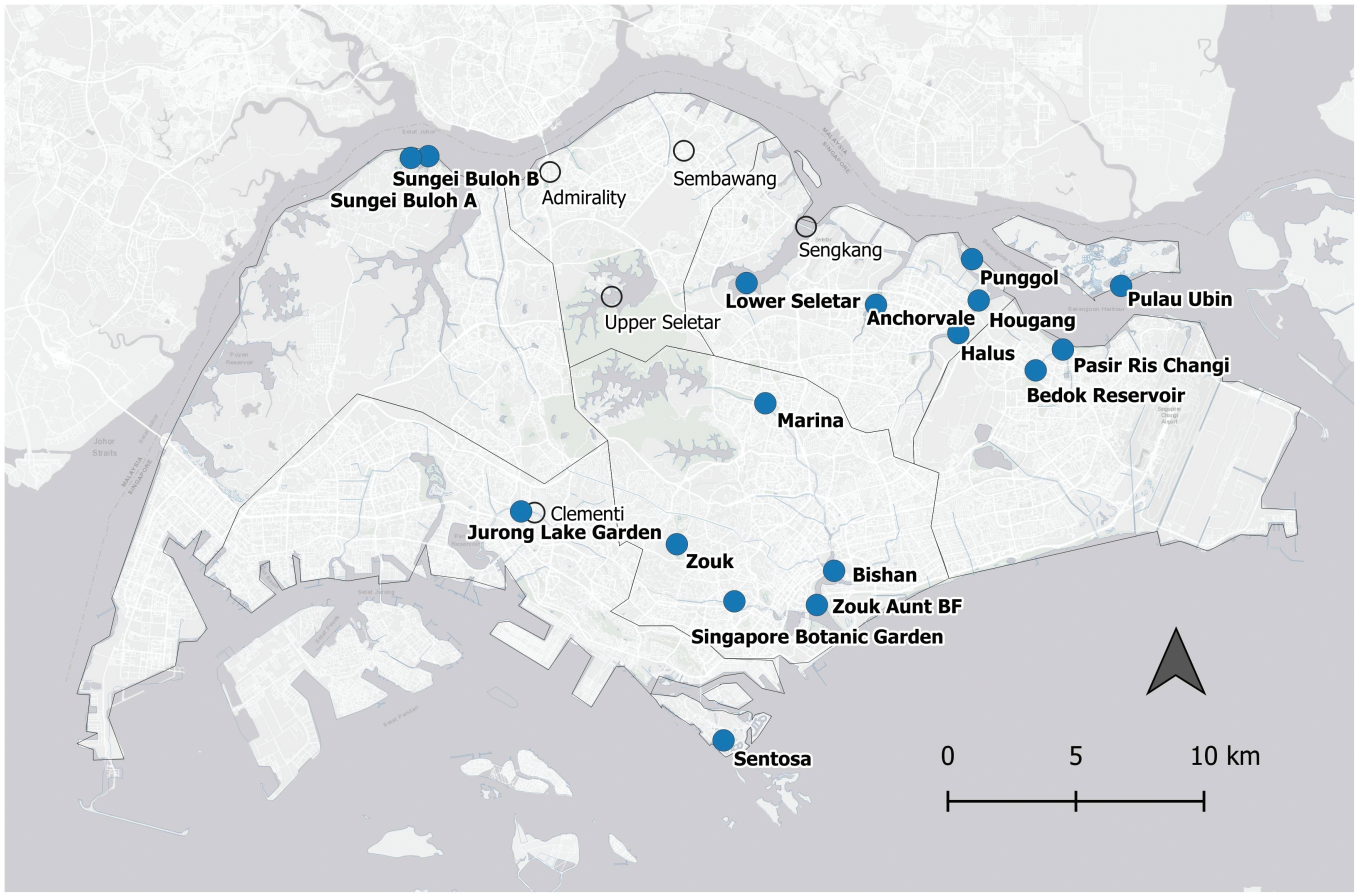
*—*Distribution of 17 smooth-coated otter groups in Singapore as of March 2021. The point locations of each group were chosen based on most visited location. Open circles indicate five other groups that had single, unverified sightings. The World Light Gray Base map was used as a base layer (Esri World Light Gray Base 2011).

During early 2021, we located 17 smooth-coated otter groups with 170 individuals (Fig. 3, Table 1). Of the 17 groups, 13 groups (126 individuals) were confirmed by both multiple observers and clear photo or video evidence, while three groups had only clear photo and video evidence by single observers, and one group had a single sighting by a reliable otter-watcher.

**Table 1.** *—*Number of smooth-coated otters, the number of adults, and number of pups in otter family groups from September 2020 to March 2021. Pups were distinguished as those with a noticeable size difference from other members of the family group as of March 2021 (‘-’ indicates that no pups were identified).

Group name	Number of smooth-coated otters		
	Total	Adult	Pup
Bishan	17	11	6
Marina	7	7	-
Singapore Botanic Garden	4	4	-
Zouk	14	7	7
Zouk Aunt BF	2	2	-
Bedok Reservoir	8	8	-
Pasir Ris Changi	7	5	2
Anchorvale	10	5	5
Halus	16	13	3
Hougang	3	3	-
Lower Seletar	10	7	3
Punggol	3	3	-
Sungei Buloh A	9	9	-
Sungei Buloh B	4	4	-
Sentosa	12	9	3
Jurong Lake Garden	20	14	6
Pulau Ubin	24	24	-
Total number	170	135	35
Percentage (%)		79.4	20.6
Average		7.9	2.1

Nine other groups (45–73 otters total) were excluded from this count (Supplementary Data SD2). Five groups of between 24 to 28 otters were omitted as each group had only one sighting that could not be verified. Four groups of between 21 to 45 otters were omitted as they could not be distinguished from other verifiable groups in the same area. Of these, it is likely that the ‘Siglap’ group (2–7 individuals) at E1 and the ‘Unknown’ group (3–8 individuals) at C1 may be duplicates, as the ‘Siglap’ group was sighted in C1.

Otter social groups ranged from 2 to 24 individuals, with an average of 10 individuals. Of the 170 individuals, 35 (20.6%) otters were distinguishable as pups in March 2021 (Table 1). There was a mean litter size of 4.4 pups in eight groups. The maximum number of pups observed was seven in the Zouk group, while the minimum was two in the Pasir Ris Changi group.

### Population distribution.—

Smooth-coated otters were distributed in urban habitats at reservoirs, waterways, urban fishponds at urban gardens, and residential and commercial sites, and in natural habitats at mangroves and coastal areas (Table 2). Thirteen groups had territories encompassing canalized or restored waterways, while four groups without waterway habitat ranged primarily along the coast. Five smooth-coated otter groups were observed foraging in fishponds in private property or urban gardens.

**Table 2.** *—*Aquatic habitats occupied by Singapore smooth-coated otter groups sorted by study zone. Habitat types are listed in order of use. Waterway habitats include canalized and naturalized watercourses. Urban fish ponds are ponds at residential and commercial sites and urban gardens. Nonurban habitats are mangroves and coastal areas.

Zone	Group	Habitat types
C1	Bishan	Reservoir, Waterway, Urban fish pond
	Marina	Reservoir, Waterway
	Singapore Botanic Garden	Waterway, Reservoir, Urban fish pond
	Zouk	Urban fish pond, Waterway, Reservoir
	Zouk Aunt and Bf	Reservoir, Waterway, Urban fish pond
E1	Bedok Reservoir	Reservoir, Waterway
	Pasir Ris Changi	Coast, Waterway
E2	Anchorvale	Waterway
	Halus	Waterway, Coast
	Hougang	Waterway, Coast
	Lower Seltar	Reservoir, Waterway
	Punggol	Coast, Waterway
E4	Pulau Ubin	Coast, Mangrove
W1	Sungei Buloh A	Mangrove, Coast
	Sungei Buloh B	Mangrove, Coast
W2	Sentosa	Coast, Urban fish pond
	Jurong Lake Gardens	Waterway, Reservoir

**Fig. 4.**
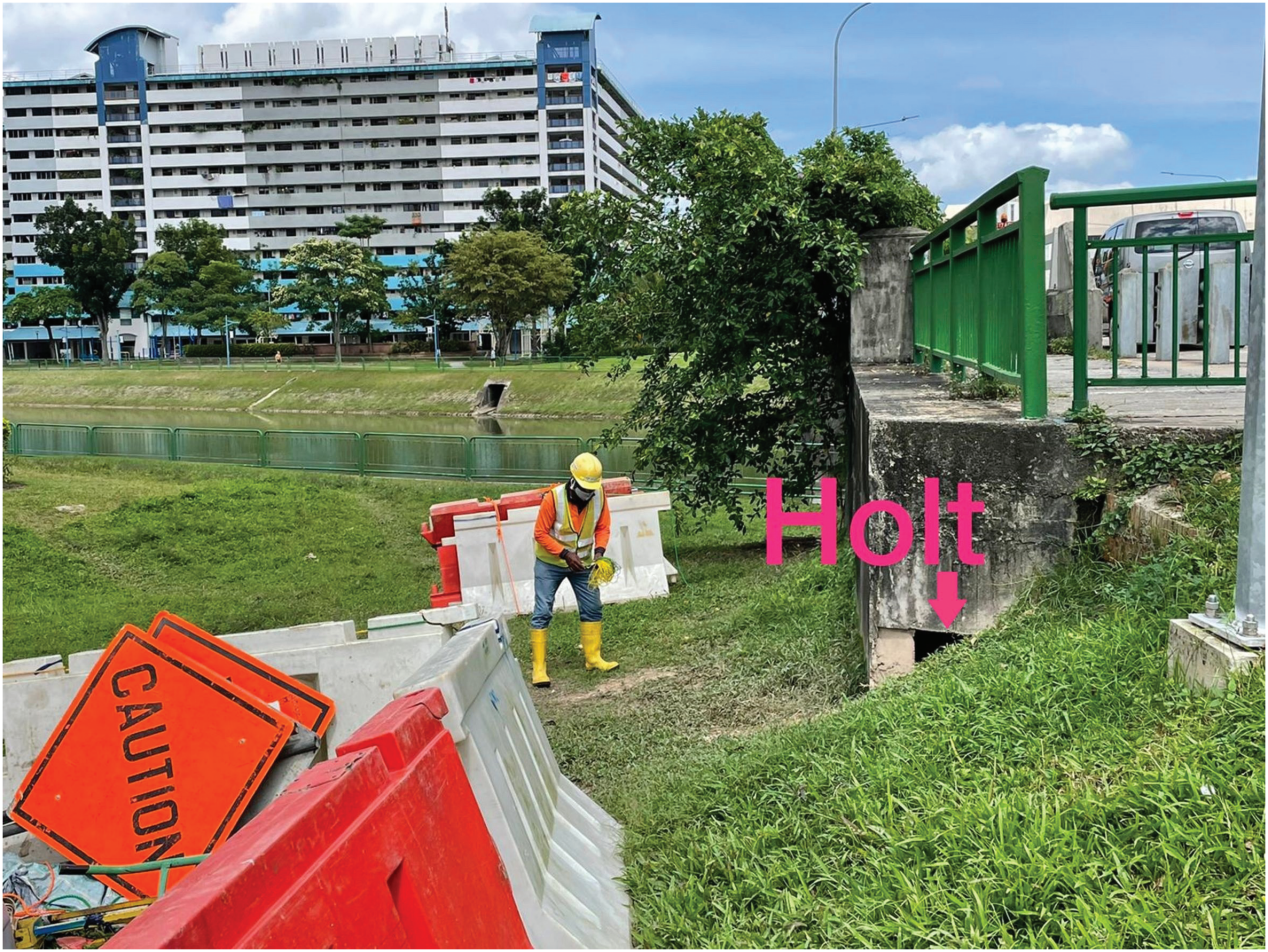
*—*Construction equipment placed adjacent to holt entrance site (labeled and marked with an arrow). Photo taken on 26 January 2021 by Marjorie Chong.

**Fig. 5.**
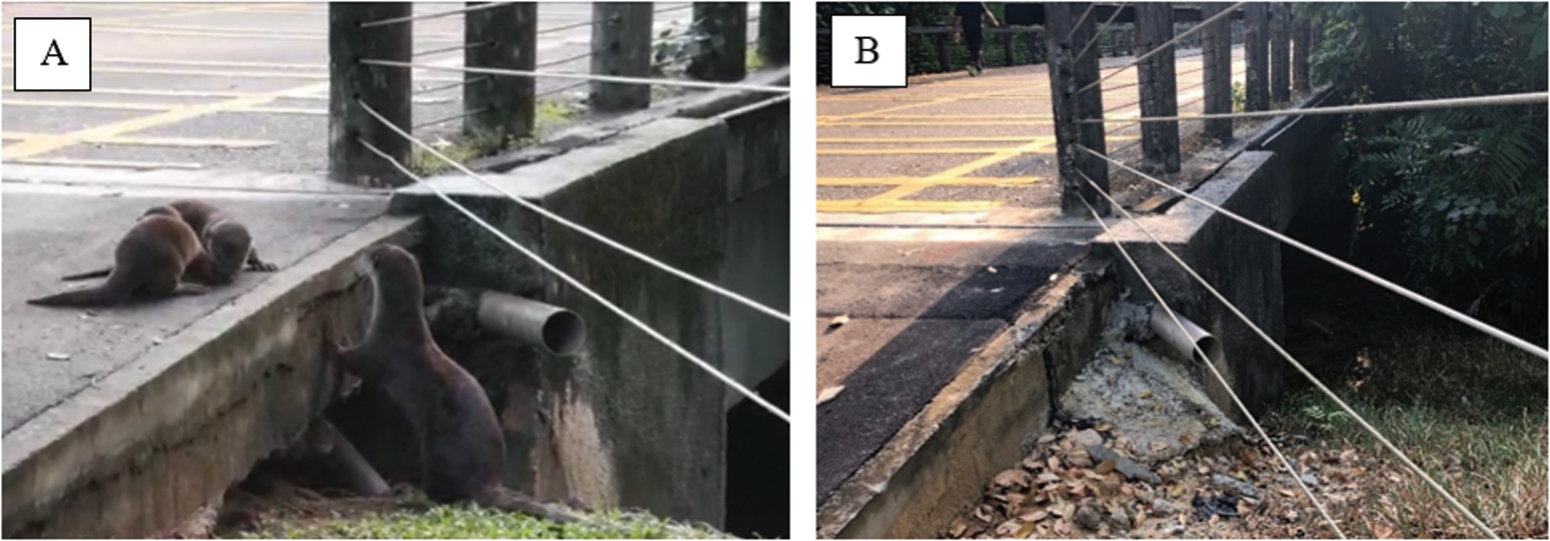
*—*(A) Smooth-coated otters using a natal holt under a bridge. Photo taken on 19 December 2019 by EP Ang. (B) The same holt site sealed using concrete. Photo taken on 18 February 2021 by AS.

Smooth-coated otters in urban Singapore created holts under built structures that can be subject to disturbances from urban development (Supplementary Data SD3). Construction activities near holts can limit smooth-coated otter access to holts, which was observed at Boon Keng and the Chinese Garden (Fig. 4). Construction at dams can block access to foraging grounds, which occurred at the Bedok Reservoir. Additionally, a smooth-coated otter holt under a bridge at Pasir Ris was filled in, preventing use of the holt (Fig. 5).

### Intergroup dynamics in the Central Watershed.—

**Fig. 6.**
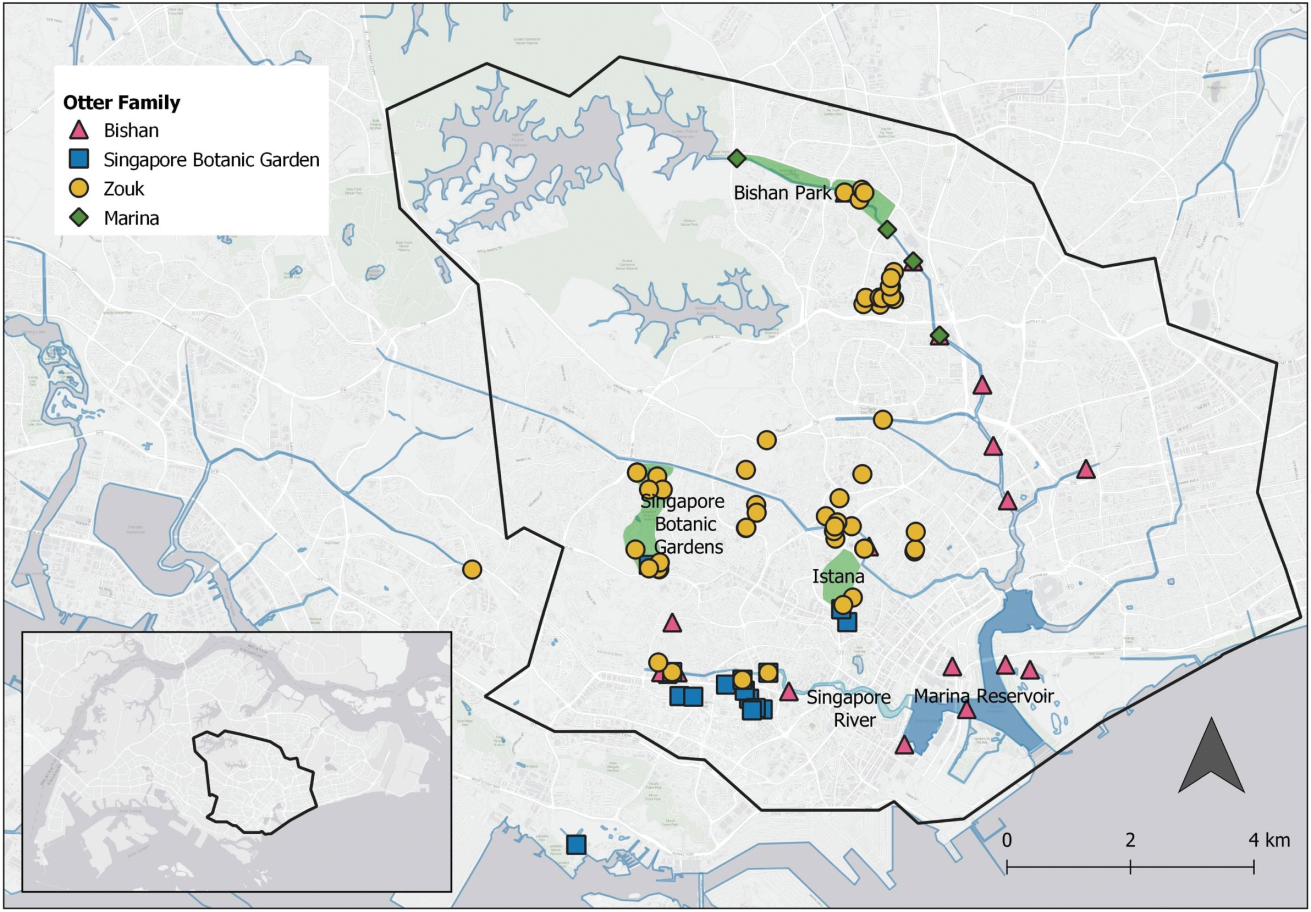
*—*Sightings of Bishan, Marina, Singapore Botanic Garden, and Zouk smooth-coated otter groups from 1 May to 1 September 2020 within the Central Watershed (C1; black border) and surrounding environs. The World Light Gray Base map was used as a base layer (Esri World Light Gray Base 2011).

Between 1 January 2020 to 1 August 2020, four groups were documented within the Central Watershed (Fig. 6). The Bishan group maintained a core territory at the Marina Reservoir and made occasional movements into the Bishan-Ang Mo Kio Park and the Singapore River at the peripheries of their home range. The Singapore Botanic Garden group occupied the Singapore River and the Marina group occupied the Bishan-Ang Mo Kio Park. The Zouk group moved largely outside main rivers and canals, traveling between waterways in residential, commercial, and green spaces, and made temporary forays into the Singapore River and the Bishan-Ang Mo Kio Park in the Kallang River.

We documented 19 interactions between the four smooth-coated otter groups, of which 12 interactions were documented by video recordings (Table 3). Smooth-coated otter conflicts occurred at the Singapore River, Bishan-Ang Mo Kio Park, and within the Singapore Botanic Gardens. Following 11 of the 16 interactions at waterways, at least one group moved away from the fight location within 3 days into urban residential, commercial, and green areas referred to in this study as the urban matrix (Table 4). Following five of these interactions, smooth-coated otter groups were sighted at Singapore Botanic Gardens.

**Table 3.** *—*Total number of territorial interactions among the Bishan, Marina, Singapore Botanic Garden (SBG), and Zouk smooth-coated otter groups in the Central Watershed recorded by otter-watchers between 1 May and 1 September 2020. The interactions are classified into those at waterways and those within the urban matrix (area surrounding waterway habitats). We recorded when otters were sighted at the urban matrix within 3 days of interactions at waterways.

Location of interaction	Bishan and SBG	Bishan and Marina	SBG and Zouk	Marina and Zouk	Total
Total Interactions	6	4	8	1	19
Interactions in waterway	5	4	6	1	16
Interactions in urban matrix	1	0	2	0	3
Sightings in the urban matrix following interactions at waterways	5	0	5	1	11

**Table 4.** *—*Number of agonistic interactions between smooth-coated otter families (Bishan, Singapore Botanic Garden [SBG], Marina, and Zouk) at each aggression level and resulting otter family that moved away from fight location. Aggression level was determined based on analysis of video recordings received of 12 interactions that occurred from 1 May 2020 to 1 September 2020.

Interacting otter groups	Withdraw	Vocalize	Escalate
Bishan and SBG		3	
Bishan and Marina		1	2
SBG and Zouk	3		2
Marina and Zouk			1

All interactions between Bishan and other otter groups involved vocalization or escalation. Zouk was the only otter group to display withdrawal; on three occasions the Zouk group approached the Singapore Botanic Garden group and withdrew without vocalization or escalation (Table 4).

### Causes of death for smooth-coated otters.—

A total of 35 smooth-coated otter mortalities were reported by the OWG between January 2019 and August 2021 with 11, 15, and 9 deaths in 2019, 2020, and during January to August 2021, respectively (Table 5, Supplementary Data SD4). The sex of 65.7% (*n* = 23) of smooth-coated otter carcasses could be determined, of which 16 (69.6%) were male. Of the 82.9% (*n* = 29) of carcasses for which age could be distinguished, 41.4% (*n* = 12) were pups and 58.6% (*n* = 17) were adults. The cause of death of eight smooth-coated otters could not be verified due to carcass decomposition or inability to retrieve carcasses. Necropsies were conducted on 27 individuals—10 in 2019, 10 in 2020, and seven in 2021.

**Table 5.** *—*Causes of mortality for 35 smooth-coated otter deaths reported by the Otter Working Group from January 2019 to August 2021. Causes of mortality were determined based on necropsy by the Mandai Wildlife Group when carcasses could be retrieved and were not significantly decomposed. When carcasses could not be retrieved, causes of mortality were either determined based on circumstances of finding or classified as ‘Unknown.’

	Cause of mortality	Total	2019	2020	2021
Human-caused mortality	Roadkill	17	6	7	4
	Fish hook	1	0	1	0
Natural mortality	Infection of unknown cause	3	2	1	0
	Intraspecific conflict^a^	6	2	1	3
	Congenital issue^a^	2	0	2	0
	Parental neglect^a^	2	1	0	1
	Unknown	4	0	3	1
Total		35	11	15	9

^a^Four smooth-coated otters were euthanized following diagnosis of congenital issues (two otters), intraspecific conflict (one otter), or pup abandonment (one otter).

Vehicle collisions were identified as the cause of death for 51.8% (*n* = 14) of the 27 carcasses for which the cause of death was verified. Of these, three otters were presumed to be roadkill as their carcasses were photographed on roads but could not be retrieved. Road-killed otters were found in zones E1, E2, and C1 (Supplementary Data SD5). Four roadkills occurred on roads that crossed a waterway, two of which occurred at the Yishun Dam, a five-lane road on a barrage separating the coast from an inland reservoir. Five roadkills occurred on roads adjacent to waterways and six roadkills occurred inland, away from waterways. The specific locations of two roadkills could not be determined.

Conflicts between conspecific groups were verified to have led to five smooth-coated otter deaths, of which three were pups. Nine of the 12 verified causes of mortality to smooth-coated otter pups were natural causes of mortality and three were human-caused mortality. Many pups were presumed to have died as they were sighted with family groups but later disappeared (nine and five pups in 2019 and 2020, respectively). Two cases of parental neglect were observed. In 2019, one Zouk otter pup was left unattended by the group and the cause of death was determined as starvation. In 2021, one pup was left unattended by the Bishan family group at the Kallang River, when the group shifted to a new holt without the pup. Despite two OWG attempts over 3 days to reunite the pup with the family group, the pup was ignored and the family moved to a different holt. Ultimately, the pup was euthanized.

## Discussion

Smooth-coated otter populations in Singapore have increased since their near extirpation during the 1970s and the 1980s (Sivasothi and Nor 1994; Theng and Sivasothi 2016; Khoo and Sivasothi 2018b). This increase in smooth-coated otter numbers is encouraging considering their conservation status of ‘Endangered’ in the Singapore Red Data Book (National Parks Board 2021) and ‘Vulnerable’ in the IUCN Red List (2021). Smooth-coated otter residence in urban waterways and pond habitats indicate their adaptability to urbanized landscapes. However, territorial interactions at urban waterways may encourage movement of groups into the urban matrix where vehicle collisions dominate threats to otter survival.

### Population abundance.—

Smooth-coated otter abundance has increased by a minimum of 91 individuals over 4 years. In 2017, 79 otters were recorded; and in 2021, we counted a minimum of 170 otters. Seven groups identified by Khoo and Sivasothi (2018b) were observed in this study (Supplementary Data SD6). Given that the locations surveyed by Khoo and Sivasothi (2018b) and this study are similar, it is likely that the increase in the number of smooth-coated otters is due to population expansion. Otter population recoveries have been documented in multiple otter species for which targeted efforts are implemented to support the conservation of species as seen in giant river otter (*Pteronura brasiliensis*) populations in northeast Peru (Uscamaita and Bodmer 2009) and Eurasian otter (*Lutra lutra*) populations in the United Kingdom (Mason and Macdonald 2004). Thus, otter population recoveries are possible with efforts to conserve otter habitats and prey and enforce wildlife laws.

We expect that our results represent an underestimate of the true otter abundance, as was also true for Khoo and Sivasothi (2018b). We did not sample the inaccessible areas of Tuas, Jurong Island, and Pulau Tekong; there were limited records of smooth-coated otters from the Western Water Catchment and parts of northern Singapore; and lone smooth-coated otters were excluded. All three otter groups sighted in the north of Singapore (E3) were excluded from the final count as these were single, unverifiable records. The smooth-coated otter population estimate will likely increase with increased research effort in the northern and western regions of Singapore. It is unlikely there will be a population increase in the central (C1) and eastern (E2) regions of Singapore where all main waterways are occupied by more than one otter group and large groups are present (C1, two groups ≥ 10 individuals; E2, three groups ≥ 10).

### Population structure.—

We observed the largest smooth-coated otter groups to date, with groups of 16, 17, 20, and 24 otters. Globally, the previous largest group of 16 was recorded in 2012 at the Corbett Tiger Reserve, India (Nawab and Hussain 2012). In 2012, the largest group in Singapore was 13 (Theng and Sivasothi 2016); and in 2017, the largest group was 14 (Khoo and Sivasothi 2018b). The large group sizes recorded in this study are outside the range of sizes for Asian small-clawed otters (*Aonyx cinereus*), which range from 1 to 12 individuals (Aziz 2018). However, similar group sizes have been recorded for giant river otters with up to 20 individuals per group (Duplaix 1980).

Large group sizes of 16, 17, and 20 were observed following pup emergence, and all three family groups had pup numbers greater than average. While the resolution of data prevented distinguishing pups in the group of 24, it is possible that the large group size is due to recently born pups. Smooth-coated otter group size increases during pup emergence but decreases following movement away from the natal area, either due to dispersal from the family or mortality (Khoo and Sivasothi 2018a). As such, the large groups observed in this study may decrease in size during postnatal home range expansion.

The litter sizes observed in this study are within the range of those previously observed in Singapore (Khoo and Sivasothi 2018b). It is likely that the pup abandoned by the Bishan group in December 2020 was from a different litter than that observed in November 2020, given marked size differences. While the numbers of breeding females per group were not recorded in this study, it is postulated that the abandoned pup was from a different breeding mother. Multiple reproductive females within a group were observed in the Bishan group in 2017 (Bungum et al. 2022).

The large number of individuals within these groups are due to multiple litters remaining with parental otters without dispersal. Smooth-coated otters display flexibility in social structures. The lack of dispersal can be explained as improving survival in poor ecological conditions and social benefits from the acquisition of experience and provision of support in sibling care (Lodé et al. 2021). In Singapore, one explanation for the limited dispersal of juveniles could be the lack of unoccupied habitats; the habitat saturation hypothesis suggests juveniles delay dispersal in these scenarios (Koenig et al. 1992). Another explanation for increased group sizes could be that larger groups can provide protection against conspecific threats. For example, the Bishan group was dominant in the Central Watershed when Bishan numbers were 13; and Zouk, Singapore Botanic Garden, and Marina group numbers were 11, 7, and 11, respectively. As group-living in otters is promoted by food availability (Lodé et al. 2021), the large groups in Singapore at Pulau Ubin, Ulu Pandan, and the Central Watershed indicate that there are abundant, replenishing resources available to host large smooth-coated otter groups within those areas.

### Population distribution.—

There has been a gradual movement of smooth-coated otter populations from mangrove and coastal areas of Singapore into reservoir habitats and waterways (Theng and Sivasothi 2016; Khoo and Lee 2020). Smooth-coated otter groups were rare in the Central Watershed in 2015 (Theng and Sivasothi 2016), but two groups were resident and raising pups in 2017 (Khoo and Sivasothi 2018b). Now, five groups occupy the Central Watershed. In addition, the Zouk and Singapore Botanic Garden groups occupy inland sites including ponds within urban gardens and residential and commercial buildings in the city center. Both the Zouk family and the Singapore Botanic Garden group navigate roads to move between waterways and highly human-dominated sites. These nomadic movements may arise among the Zouk group due to unsuccessful attempts of the group to expand home range into waterways.

Most groups had territories in human-dominated landscapes, with otters navigating reservoirs and waterways. Urban habitat use by smooth-coated otters has been documented in the Inner Gulf of Thailand; however, smooth-coated otters there avoid human disturbance (Kamjing et al. 2017). When using human-made structures to create holts and forage, smooth-coated otters are subject to disturbances such as the infilling and blocked access to holts and foraging sites observed in this study. Smooth-coated otters have high holt fidelity, using the same holts across multiple litters (Anoop and Hussain 2004). Similarly, the seven groups previously identified by Khoo and Sivasothi (2018b) were observed occupying the same areas in this study. The infilling of holts at one site reduces the number of available holts across their home ranges and may increase energy expenditure during the creation of new holts.

### Intergroup dynamics.—

Four smooth-coated otter groups in the Central Watershed displayed spatial and temporal segregation, occupying different core habitat areas with overlapping peripheries. When groups co-occurred, territorial interactions involving vocalizations and escalation to physical fights were observed on all but three occasions when the Zouk group withdrew.

Territorial interactions occurred both at waterways and on land, adjacent to known holt sites. Following territorial interactions at waterways, smooth-coated otter groups that moved away were sighted at waterway sites away from the fight location or within the urban matrix. The spatial and temporal avoidance between otter groups in space-limited areas has also been observed in Eurasian otters in Northern Scotland (Jenkins 1980).

While it was expected that an increase in interspecies interactions would result in higher smooth-coated otter mortality caused by conflicts, none of the 19 interactions in this study directly caused smooth-coated otter death. It is possible that smooth-coated otter groups may prevent conflict occurrence and aggravation by avoidance, as observed in three occasions when the Zouk family approached the Singapore Botanic Garden group and withdrew without contest. Eurasian otters in Sweden appear to prioritize avoidance compared to pursuits (Erlinge 1968). Similarly, conflict avoidance through vocalizations and scent-marking has been documented among giant river otter groups (Leuchtenberger et al. 2015; Mumm and Knörnschild 2017). Conflict avoidance was postulated to occur to avoid the high costs of aggressive encounters, such as infanticide and adult otter injury or death.

As smooth-coated otter population density increases, territorial conflicts may be increasingly common as multiple groups are within reasonable proximity. Intraspecific conflicts were also observed between the Anchorvale and Halus groups in E2, where a total of five groups reside. Spatiotemporal avoidance behaviors at these waterways may influence movement of smooth-coated otters into residential areas surrounding waterways. Hence, it can be valuable to prepare for potential human–wildlife conflict at otter-dense waterways such as those in E2 and C1.

### Causes of mortality.—

As expected with population increases and saturation of waterways, smooth-coated otters are navigating the urban matrix in search of suitable habitat, increasing the chance for road-traffic caused mortality. Vehicle collisions were the main cause of smooth-coated otter mortality in Singapore. Unsurprisingly, most roadkills were located on roads that crossed or were adjacent to waterways. Similarly, Eurasian otter roadkills in Britain mainly occurred at roads that crossed a watercourse (Philcox et al. 1999). The association between roadkills and river proximity has also been observed for other wildlife that are dependent on aquatic and terrestrial landscapes such as capybaras (*Hydrochoerus hydrochaeris*; Bueno et al. 2013) and amphibians (Sillero et al. 2019).

As previously discussed (Kruuk and Conroy 1991; Poledník et al. 2011), with opportunistically sourced data, there is a higher probability of encountering otter carcasses adjacent to roads compared to finding carcasses of animals that died of natural causes such as old age or disease. This possible detection bias can skew the proportion of deaths attributed toward nonnatural causes. Given such biases, we are unable to comment on the relative importance of vehicle collision as a cause of mortality for otters in Singapore. However, it is still worth evaluating measures to reduce smooth-coated otter deaths by vehicle collision. Methods to reduce roadkills in Singapore include the creation of culverts, the conversion of multilane roads to a single lane with a park connector, and the use of camera detection to alert drivers, such as a system installed in roadkill-prone areas near a Nature Reserve in Singapore (Tan 2019b). Such methods can be evaluated and applied to identified roadkill hotspots.

We observed two cases of parental neglect in this study, when adults left pups unattended and did not feed them. Pup abandonment has been documented in sea otters (*Enhydra lutris*) and Eurasian otters and was postulated to occur when mothers are resource-limited or die (Garshelis and Garshelis 1987; Poledník et al. 2011). However, in this study, pup abandonment occurred despite the presence of the mother and the holt-adjacent waterway with abundant exotic fish. Fourteen pups were presumed to have died as they were initially seen with their family groups, but then disappeared. Hence, pup deaths may have been from more causes than we detected.

Fishing activities along waterways have the potential to injure smooth-coated otters. The first case of human-caused death of a smooth-coated otter was recorded in Singapore in 2017 when an otter became entangled in an illegal fishing trap (Khoo and Sivasothi 2018a). In 2018, one smooth-coated otter was found dead in an illegal fishing trap with another carcass nearby (Ng 2018). Fishing activities in legal fishing zones can also lead to otter injuries and death. One 4-month-old pup was found dead with a fishing hook perforating its esophagus and the OWG received two other reports of otters injured by fishhooks (Supplementary Data SD4). These deaths could be avoided by reducing illegal fishing practices and educating anglers in the safe disposal of fishing equipment.

During this study, we observed three reports of interactions between feral dogs and smooth-coated otters in the eastern region of Singapore and one in the northern region (Supplementary Data SD7). The population size of feral dogs in Singapore is unknown, but 3,116 dogs were trapped for the Trap-Neuter-Release-Manage program in 2019–2021 (National Parks Board 2022). Only one of these interactions involved direct attack of an otter pup by a feral dog. In the remaining interactions, smooth-coated otters were able to deter or navigate away from feral dog packs. Feral dogs pose threats to smooth-coated otters in Pakistan and India, impacting smooth-coated otter presence (Anoop and Hussain 2004; Khan et al. 2009). In Singapore, it appears that feral dogs pose a significant threat to smooth-coated otter pups but only a minimal threat to adult smooth-coated otters that are able to navigate away from feral dogs.

### Recommendations for annual otter census.—

Smooth-coated otter populations continue to change, with new pups observed in four groups after we ended sampling in March 2021 and with reports of smooth-coated otters in areas excluded from this study (Supplementary Data SD8). The network of otter-watchers established for this study who were willing to participate in a rigorous evidence-based census indicates the potential to conduct an annual otter census. Other citizen-science censuses have been carried out successfully to monitor Eurasian otters in England and Sweden (Kent 2015; Loso and Roos 2019) and North American river otters (*Lontra canadensis*) in Northern California (Black et al. 2016). With the inclusion of more otter-watchers in such collaborative efforts, surveys of the inaccessible and restricted sites of the past two surveys could be conducted to establish an understanding of smooth-coated otter populations in the Western and Northern regions of Singapore.

### Conservation implications.—

The recovery of smooth-coated otter populations observed in Singapore can inform strategies for the conservation and management of this species in other urban areas. In Coimbatore, India and in Kuala Lumpur, Malaysia, smooth-coated otters have been recently observed in urban waterways and gardens (Choong 2019; Jun 2019; Thomas 2021). Population recoveries appear possible with suitable habitats and abundant prey. As populations increase, surveys that include data on intraspecies interactions and movements may highlight spatiotemporal segregation. Studying the causes of smooth-coated otter mortality can enable targeted approaches to mitigate threats.

While the increase in smooth-coated otter populations is welcomed given their poor conservation status globally, threats to smooth-coated otters and human–otter conflicts need to be managed to prevent future population declines.

## Supplementary Data

Supplementary data are available at *Journal of Mammalogy* online.

**Supplementary Data SD1.**—The citizen scientists who provided smooth-coated otter data.

**Supplementary Data SD2.**—Evidence used to include or exclude observations of smooth-coated otter groups.

**Supplementary Data SD3.**—Human disturbance to smooth-coated otter holts and foraging areas.

**Supplementary Data SD4.**—Mortality details for smooth-coated otters.

**Supplementary Data SD5.**—Locations of smooth-coated otter roadkills.

**Supplementary Data SD6.**—Comparison of smooth-coated otter groups in 2017 and 2021.

**Supplementary Data SD7.**—Interactions between smooth-coated otters and feral dogs.

**Supplementary Data SD8.**—Changes in the number of smooth-coated otters in six groups in spring 2021.

gyad007_suppl_Supplementary_Data_S1

gyad007_suppl_Supplementary_Data_S2

gyad007_suppl_Supplementary_Data_S3

gyad007_suppl_Supplementary_Data_S4

gyad007_suppl_Supplementary_Data_S5

gyad007_suppl_Supplementary_Data_S6

gyad007_suppl_Supplementary_Data_S7

gyad007_suppl_Supplementary_Data_S8
