# Supplementary material for: Population distribution and causes of mortality of smooth-coated otters, Lutrogale perspicillata, in Singapore
Source: J Mammal. 2023 Mar 1;104(3):496–508. doi: 10.1093/jmammal/gyad007 (PMC10243989; doi:10.1093/jmammal/gyad007)
Supplement: gyad007_suppl_Supplementary_Data_S1 [file gyad007_suppl_supplementary_data_s1.docx]

**Supplementary Data S1.** **—** Sources of information on Singapore smooth-coated otter (*Lutrogale perspicillata*) populations in each sampling unit. Each contacted observer was either directly known through the Otter Working Group (OWG) and Otterfriends WhatsApp group. Some observers frequent multiple study zones.

| Sampling Unit | Name | Contact Method |
| --- | --- | --- |
| C1 | Marjorie C. | OWG |
|  | Bernard S. | OWG |
|  | Jeffery T. | OWG |
|  | Jo W. | OWG |
|  | James W. | OWG^a^ |
| E1 | Evan L. | Otterfriends |
|  | Sophie B. | Otterfriends^a^ |
|  | Mickey H. | Otterfriends^a^ |
|  | EP Ang | Otterfriends^a^ |
|  | L. Ah Eng | Otterfriends |
| E2 | Marjorie C. | OWG |
|  | Sylvia L. | OWG^a^ |
|  | Jocelyn C. | OWG^a^ |
|  | Daniel C. | OWG^a^ |
|  | Haslinda M. Y. | OWG^a^ |
| E3 | - | - |
| E4 | Mei Hwang | OWG^a^ |
| W1 | Bernard S. | OWG |
| W2 | Jo W. | OWG |
|  | Christopher J. | Student Researcher |

^a^Observers contacted through mutual contacts from either the Otterfriends WhatsApp group or the OWG.
