# Supplementary material for: Population distribution and causes of mortality of smooth-coated otters, Lutrogale perspicillata, in Singapore
Source: J Mammal. 2023 Mar 1;104(3):496–508. doi: 10.1093/jmammal/gyad007 (PMC10243989; doi:10.1093/jmammal/gyad007)
Supplement: gyad007_suppl_Supplementary_Data_S2 [file gyad007_suppl_supplementary_data_s2.docx]

**Supplementary Data S2.** **—** The number of smooth-coated otters (*Lutrogale perspicillata*) in Singapore per social group from September 2020 to March 2021, evidence available and reasons for final count. Locations of groups are indicated by zones Central Watershed (C1), Eastern Watershed (E1 – E4), and the Western Watershed (W1, W2) (Public Utilities Board 2014).

| Zone | Group | No. of dates with sighting | Min group size | Max group size | Modal count | Final count | Most recent date with final count | Excluded (Y/N) | Reason for final count/exclusion |
| --- | --- | --- | --- | --- | --- | --- | --- | --- | --- |
| C1 | Bishan | 16 | 3 | 17 | 17 | 17 | 15-Jan-21 | N | Clear evidence of 17 available, most reported number. |
|  | Marina | 4 | 6 | 11 | 10 | 7 | 29-Sep-20 | N | Clear evidence of 7 available, most recently reported by a reliable otter-watcher from the OWG. Concurrent sightings with Zouk and Bishan. |
|  | SBG | 12 | 3 | 5 | 4 | 4 | 1-Mar-21 | N | Clear evidence of 4 available, most reported number. Concurrent sightings with Zouk and Bishan. |
|  | Zouk | 32 | 7 | 14 | 14 | 14 | 5-May-21 | N | Clear evidence of 14 available, most reported number. Concurrent sightings with SBG, Marina and Bishan |
|  | Zouk and and Bf | 2 | 2 | 2 | 2 | 2 | 6-Feb-21 | N | Clear evidence of 2 available, most reported number. Concurrent sightings with Bishan. |
|  | Unknown | 3 | 3 | 8 | 8, 7 |  |  | Y | Could not be distinguished from other otter groups. |
| E1 | Bedok Reservoir | 3 | 8 | 8 | 8 | 8 | 30-Oct-20 | N | Clear evidence of 8 available, most reported number. Concurrent sightings with Pasir Ris Changi. |
|  | Congo | 4 | 1 | 2 | 1,2 |  |  | Y | Most recently sighted as one individual. |
|  | Pasir Ris Changi | 17 | 3 | 7 | 7 | 7 | 12-Mar-21 | N | Clear evidence of 7 available, most reported number. Concurrent sightings with Pasir Ris Changi. |
|  | Siglap | 7 | 2 | 7 | 7 |  |  | Y | While Siglap could be distinguished from Tanah Merah, it could not be distinguished from Bedok Reservoir. |
|  | Tanah Merah | 4 | 9 | 13 | 9 |  |  | Y | While Tanah Merah could be distinguished from Siglap, it could not be distinguished from Bedok Reservoir. |
| E2 | Sengkang | 1 | 4 | 5 | 5,4 |  |  | Y | Single, unverifiable record. |
|  | Lower Seletar | 4 | 5 | 12 | 10 | 10 | 13-Feb-21 | N | Clear evidence of 10 available, most reported number. Concurrent sightings with Punggol. |
|  | Punggol | 39 | 1 | 9 | 3 | 3 | 17-Feb-21 | N | Clear evidence of 3 available, most reported number. Family history of deaths clearly documented. |
|  | Anchorvale | 15 | 5 | 10 | 10 | 10 | 13-Feb-21 | N | Clear evidence of 10 available, most reported number. Concurrent sightings with Houngang Trio, Punggol, Lower Seletar. |
|  | Halus | 15 | 3 | 16 | 14 | 16 | 20-Jan-21 | N | Clear evidence of 16 available, most recently reported number. Concurrent sightinsg with Houngang Trio, Anchorvale, Punggol. |
|  | Hougang Trio | 26 | 2 | 3 | 3 | 3 | 2-Mar-21 | N | Clear evidence of 3 available, most reported number. Concurrent sightings with Punggol, Halus, Anchorvale. |
| E3 | Admiralty | 1 | 8 | 8 | 8 |  |  | Y | Single, unverifiable record. |
|  | Sembawang | 1 | 6 | 6 | 6 |  |  | Y | Single, unverifiable record. |
|  | Upper Seletar | 1 | 2 | 2 | 2 |  |  | Y | Single, unverifiable record. |
| E4 | Pulau Ubin | 4 | 12 | 24 | 12 | 24 | 2-Mar-21 | N | Clear evidence of 24 available, most recently reported number. |
| W1 | Sungei Buloh A | 2 | 6 | 9 | 9 | 9 | 4-Mar-21 | N | Clear evidence of 9 available, most reported number. Concurrent sighting with Sungei Buloh B |
|  | Sungei Buloh B | 1 | 4 | 4 | 4 | 4 | 3-Mar-21 | N | Clear evidence of 4 available, most reported number. Concurrent sighting with Sungei Buloh A |
|  | Unknown | 1 | 1 | 1 | 1 |  |  | Y | Single otter, could not be distinguished from Sungei Buloh A and Sungei Buloh B |
| W2 | Jurong Lake Garden | 19 | 6 | 20 | 20 | 20 | 19-Feb-21 | N | Clear evidence of 20 available, most reported number |
|  | Sentosa | 5 | 4 | 12 | 10 | 12 | 28-Feb-21 | N | Clear photographic evidence of 12. Family is known to leave pups behind at holt while travelling. |
|  | Ulu Pandan | 4 | 7 | 17 | 7,8 |  |  | Y | Ulu Pandan family could not be distinguished from Jurong Lake Gardens as there were no concurrent sightings of the family groups |
|  | Clementi | 1 | 5 | 5 | 5 |  |  | Y | Single, unverifiable record. |
