# Supplementary material for: Population distribution and causes of mortality of smooth-coated otters, Lutrogale perspicillata, in Singapore
Source: J Mammal. 2023 Mar 1;104(3):496–508. doi: 10.1093/jmammal/gyad007 (PMC10243989; doi:10.1093/jmammal/gyad007)
Supplement: gyad007_suppl_Supplementary_Data_S3 [file gyad007_suppl_supplementary_data_s3.docx]

**Supplementary Data S3.** **—** Human-caused disturbances due to urban development observed at three smooth-coated otter (*Lutrogale perspicillata*) holts and two foraging grounds in Singapore.

| Holts and foraging grounds with disturbance | Disturbance | Groups affected |
| --- | --- | --- |
| Boon Keng Holt | Construction equipment blocking holt | Bishan |
| Pasir Ris Bridge Holt | Infilling of holt | Pasir Ris Changi |
| Chinese Garden Holt | Construction at holt | Jurong Lake Gardens |
| Indus Holt | Infilling of holt | Bishan, Singapore Botanic Garden, Zouk |
| Singapore River Foraging Ground | Construction at park connector | Bishan, Singapore Botanic Garden, Zouk |
| Bedok Reservoir Foraging Ground | Construction at river | Bedok Reservoir |
