# Supplementary material for: Population distribution and causes of mortality of smooth-coated otters, Lutrogale perspicillata, in Singapore
Source: J Mammal. 2023 Mar 1;104(3):496–508. doi: 10.1093/jmammal/gyad007 (PMC10243989; doi:10.1093/jmammal/gyad007)
Supplement: gyad007_suppl_Supplementary_Data_S4 [file gyad007_suppl_supplementary_data_s4.docx]

**Supplementary Data S4.** **—** Smooth-coated otter (*Lutrogale perspicillata*) mortality records for January 2019 to August 2021 based on post-mortem data from Wildlife Reserves Singapore and carcasses observed by the Otter Working Group. The group, age, sex, location of death and causes of mortality are included where information was available. Some otter carcasses could not be retrieved for post-mortem.

| Date | Age | Sex | Location | Plausible cause of death | Post-mortem (Y/N) | Group |
| --- | --- | --- | --- | --- | --- | --- |
| 11-Mar-19 | A | F | Bedok Reservoir | Roadkill | Y |  |
| 13-Mar-19 | P |  | Braddell | Infection | Y |  |
| Apr-19 |  |  | Sengkang | Roadkill | N |  |
| 30-May-19 | P |  |  | Intraspecific conflict | Y |  |
| 30-May-19 | P |  |  | Intraspecific conflict | Y |  |
| 30-May-19 | A | F | Yishun | Roadkill | Y |  |
| 7-Jun-19 | P | M |  | Roadkill | Y |  |
| 10-Jun-19 | P |  | East Coast Park | Roadkill | Y |  |
| 19-Jul-19 | A | M | Yishun Pond | Infection | Y |  |
| 25-Dec-19 | P | M | Singapore Botanic Gardens | Starvation, Parent neglect | Y | Zouk |
| 26-Dec-19 |  | M | Braddell | Roadkill | Y |  |
| 20-Jan-20 | P | M |  | Euthanised, Congenital deformity (Hydrocephalus) | Y | Zouk |
| 20-Jan-20 | P | M |  | Euthanised, Congenital deformity (Hydrocephalus) | Y | Zouk |
| 28-Jan-20 | P |  | Under bridge of flyover from Punggol to Lor Halus |  | N |  |
| 7-Feb-20 | A | M | 608 Telok Blangah Road, Skyline Residences | Roadkill | Y |  |
| 2-May-20 | A |  | Blk 125 Aljuned Road Pelton canal |  | N |  |
| 16-Jun-20 | A |  | Dunearn Road before turf city | Roadkill | N |  |
| 24-Jun-20 | A | F | Kallang Basin | Intraspecific conflict | Y |  |
| 21-Jul-20 |  |  | Ulu Pandan River |  | N (monitor lizard feeding) |  |
| 9-Aug-20 | A | F | Coney Island Beach | Infection | Y | Punggol |
| 1-Sep-20 | A | M | Tampines North Drive 1 Canal | Roadkill | Y |  |
| 5-Sep-20 | P | M | Punggol Marina | Infection due to perforation of oesophagus by fish hook^a^ | Y | Punggol |
| 20-Sep-20 | A | M | Ayer Rajah Expressway | Roadkill | Y | Singapore Botanic Garden |
| 20-Sep-20 | A | M | Ayer Rajah Expressway | Roadkill | Y | Singapore Botanic Garden |
| 21-Oct-20 |  |  | Along Seletar West Link towards Yishun dam after U-turn, middle lane | Roadkill | N |  |
| 21-Dec-20 | A | M | Yishun Dam | Roadkill | N |  |
| 7-Jan-21 | P | F | Abandoned at Republic Holt | Euthanasia, Parent neglect | Y |  |
| 11-Jan-21 | A |  | Pandan Reservoir |  | N |  |
| 18-Jan-21 |  |  | Sengakng Way | Intraspecific conflict | N (monitor lizard feeding) | Anchorvale |
| 16-Apr-21 | P | F | Changi Airport Aerodrome | Euthanasia, Intraspecific conflict | Y |  |
| 23-May-21 | A | M | Holland Road | Roadkill | Y | Zouk |
| 16-Jul-21 | A | M | 9A Lock Road | Roadkill | Y | Zouk |
| 16-Jul-21 | A | F | 9A Lock Road | Roadkill | Y | Zouk |
| 28-Jul-21 |  | M | Esplanade stage | Intraspecific conflict | Y |  |
| 10-Aug-21 | A | M | KPE/Punggol (Buangkok East Dr) | Died during post-mortem, Roadkill | Y | Halus |

^a^Two other otters were non-lethally affected by fish hooks. On 8 Sep 2020, a Zouk group adult otter was observed with a fish hook on its body at Singapore River. On 2 Aug 2021, an otter was observed stuck to a fishing line at Siglap canal.
