# Supplementary material for: Population distribution and causes of mortality of smooth-coated otters, Lutrogale perspicillata, in Singapore
Source: J Mammal. 2023 Mar 1;104(3):496–508. doi: 10.1093/jmammal/gyad007 (PMC10243989; doi:10.1093/jmammal/gyad007)
Supplement: gyad007_suppl_Supplementary_Data_S5 [file gyad007_suppl_supplementary_data_s5.docx]

**Supplementary Data S5.** **—** Location of smooth-coated otter (*Lutrogale perspicillata*) roadkills from January 2019 to August 2021 in study zones and presence of nearby waterway. Roadkills that are found away from waterways are recorded as inland. Study zones are the Central Watershed (C1), Eastern Watershed (E1 – E4), and the Western Watershed (W1, W2) (Public Utilities Board 2014).

| Location of roadkill | Study zone | Presence of nearby waterway |
| --- | --- | --- |
| **2019** | | |
| Bedok Reservoir | E1 | Adjacent to waterway |
| Sengkang, near Tebing lane | E2 | Adjacent to waterway |
| Yishun | E2 | Unknown |
| Unknown |  | Unknown |
| East Coast Park | E1 | Adjacent to waterway |
| Braddell Road | C1 | Crosses waterway |
| **2020** | | |
| Dunearn Road | C1 | Adjacent to waterway |
| 608 Telok Blangah Road, Skyline Residences | W2 | Inland |
| Tampines North Drive 1 | E1 | Adjacent to waterway |
| Ayer Rajah Expressway (2 roadkills) | C1 | Inland |
| Yishun Dam | E2 | Crosses waterway |
| **2021** |  |  |
| Holland Road | C1 | Inland |
| 9A Lock Road (2 roadkills) | C1 | Inland |
| KPE/Punggol Buangkok East Drive | E2 | Crosses waterway |
