# Supplementary material for: Population distribution and causes of mortality of smooth-coated otters, Lutrogale perspicillata, in Singapore
Source: J Mammal. 2023 Mar 1;104(3):496–508. doi: 10.1093/jmammal/gyad007 (PMC10243989; doi:10.1093/jmammal/gyad007)
Supplement: gyad007_suppl_Supplementary_Data_S6 [file gyad007_suppl_supplementary_data_s6.docx]

**Supplementary Data S6.** **—** The number, names, and structure of smooth-coated otter groups (*Lutrogale perspicillata*) in each study zone in 2017 (Khoo and Sivasothi 2018b) and in the current study from September 2020 to March 2021. Group names are accompanied by the number of smooth-coated otters within each group. Study zones are the Central Watershed (C1), Eastern Watershed (E1 – E4), and the Western Watershed (W1, W2) (Public Utilities Board 2014).

| Study Zone | Groups in 2017 (Khoo and Sivasothi 2018) | Groups in 2021 |
| --- | --- | --- |
| C1 | 2 (Marina: 7, Bishan: 13) | 5 (Marina: 7, Bishan: 17, Zouk: 14, Singapore Botanic Garden: 4, Zouk Aunt BF: 2) |
| E1 | 2 (Pasir Ris: 7, Tanah Merah: 9) | 2 (Pasir Ris Changi: 7, Bedok Reservoir: 8) |
| E2 | 3 (Lower Seletar: 7, Sengkang: 5, Coney Island-Serangoon Reservoir: 10) | 5 (Lower Seletar: 10, Punggol: 3, Anchorvale: 10, Halus: 16, Hougang: 3) |
| E3 | 0 | 0 |
| E4 | 0 | 1 (PUB24) |
| W1 | 1 (Sungei Buloh: 4) | 2 (Sungei Buloh A: 9, Sungei Buloh B: 4) |
| W2 | 3 (Jurong Lake: 2, Ulu Pandan: 8, Sentosa: 7) | 2 (Jurong Lake: 20, Sentosa: 12) |
