# Supplementary material for: Population distribution and causes of mortality of smooth-coated otters, Lutrogale perspicillata, in Singapore
Source: J Mammal. 2023 Mar 1;104(3):496–508. doi: 10.1093/jmammal/gyad007 (PMC10243989; doi:10.1093/jmammal/gyad007)
Supplement: gyad007_suppl_Supplementary_Data_S7 [file gyad007_suppl_supplementary_data_s7.docx]

**Supplementary Data S7.** **—** Interactions between smooth-coated otters (*Lutrogale perspicillata*) and feral dogs (*Canis familiaris*) in Singapore recorded from January 2019 to March 2021.

| Date | Location | Number of feral dogs | Number of smooth-coated otters | Result of interaction |
| --- | --- | --- | --- | --- |
| 19 Jun 2020 | Sembawang Park | 3 | 6 | Feral dogs chased by smooth-coated otters |
| 27 Jun 2020 | Punggol Beach | 8 | 5 pups | One pup caught by feral dog, eventual escape of smooth-coated otters |
| 28 Aug 2020 | Pasir Ris Beach | 2 | 1 | Otter escaped unharmed |
| 1 Feb 2021 | Pasir Ris Beach | 4 | 1 | Otter escaped unharmed |
