# Supplementary material for: Population distribution and causes of mortality of smooth-coated otters, Lutrogale perspicillata, in Singapore
Source: J Mammal. 2023 Mar 1;104(3):496–508. doi: 10.1093/jmammal/gyad007 (PMC10243989; doi:10.1093/jmammal/gyad007)
Supplement: gyad007_suppl_Supplementary_Data_S8 [file gyad007_suppl_supplementary_data_s8.docx]

**Supplementary Data S8.** **—** Changes in the number of smooth-coated otters (*Lutrogale perspicillata*) in six groups from 5 March to 26 April 2021. Changes refer to sightings of new litters or sightings of new groups in Singapore.

| Group name | Change in smooth-coated otter numbers | | Date of change | Total smooth-coated otters in group as of 26 April 2021 |
| --- | --- | --- | --- | --- |
| Bishan | | 5 pups born | 7 Mar 2021 | 22 |
| Jurong Lake Gardens | | 3 pups born | 1 Apr 2021 | 23 |
| Aquarius | | 3 adults, 3 pups | 19 Mar 2021 | 6 |
| Singapore Botanic Garden | | 4 pups born | 21 Apr 2021 | 8 |
| Admiralty | | 5-6 otters | 2 Apr 2021 | 5-6 |
| Sembawang | | 11 otters | 31 Mar 2021 | 11 |
